# Outcome of Critically ill Patients Undergoing Mandatory Insulin Therapy Compared to Usual Care Insulin Therapy: Protocol for a Pilot Randomized Controlled Trial 

**DOI:** 10.2196/resprot.5912

**Published:** 2018-03-08

**Authors:** Peter J Watkinson, Vicki S Barber, J Duncan Young

**Affiliations:** ^1^ Critical Care Trials Group University of Oxford Oxford United Kingdom; ^2^ Oxford Clinical Trials Research Unit University of Oxford Oxford United Kingdom

**Keywords:** intensive care, insulin, glycaemic control

## Abstract

**Background:**

Observational and interventional studies in patients with both acute medical conditions and long-standing diabetes have shown that improved blood glucose control confers a survival advantage or reduces complication rates. Policies of “tight” glycaemic control were rapidly adopted by many general intensive care units (ICUs) worldwide in the mid 00’s, even though the results of the studies were not generalizable to mixed medical/surgical ICUs with different intravenous feeding policies.

**Objective:**

The primary objective of the study is to assess the safety of mandatory insulin infusion in critically ill patients in a general ICU setting.

**Methods:**

This protocol summarizes the rationale and design of a randomized, controlled, single-center trial investigating the effect of mandatory insulin therapy versus usual care insulin therapy for those patients admitted for a stay of longer than 48 hours. In total, 109 critically ill adults predicted to stay in intensive care for longer than 48 hours consented. The primary outcome is to determine the safety of mandatory insulin therapy in critically ill patients using the number of episodes of hypoglycaemia and hypokalaemia per unit length of stay in intensive care. Secondary outcomes include the duration of mechanical ventilation, duration of ICU and hospital stay, hospital mortality, and measures of renal, hepatic, and haematological dysfunction.

**Results:**

The project was funded in 2005 and enrolment was completed 2007. Data analysis is currently underway and the first results are expected to be submitted for publication in 2018.

**Conclusions:**

This protocol for a randomized controlled trial investigating the effect of mandatory insulin therapy should provide an answer to a key question for the management of patients in the ICU and ultimately improving outcome.

**Trial Registration:**

International Standard Randomized Controlled Trial Number ISRCTN00550641; http://www.isrctn.com/ISRCTN00550641 (Archived at WebCite: http://www.webcitation.org/6xk8NXxNv).

## Introduction

### Background

Observational and interventional studies in patients with both acute medical conditions and long-standing diabetes have shown that improved blood glucose control confers a survival advantage or reduces complication rates [1-4]. In 2001, Van den Berghe and colleagues extended this to critically ill patients with no history of diabetes when they published the results of a study of “tight” blood glucose control compared with conventional blood glucose control in Dutch intensive care unit (ICU) patients following surgery [5]. They showed a corrected relative reduction in intensive care mortality of 32% in the “tight” control group, with the benefits primarily seen in the patients who required a prolonged ICU stay. A reduced ICU length of stay and a reduction in the incidence of renal dysfunction and nosocomial infections were also seen in the “tight” glucose control patients.

Tight glycaemic control therapy necessitates increased insulin administration to achieve euglycaemia. This raises the possibility that any patient benefits may be a result of the insulin delivered rather than the glycaemic control attained, and that further improvements in patient outcomes might be possible with increased insulin administration [6]. However, intensive insulin therapy is a complex intervention. The effects depend upon the protocol used, feeding strategies, staff training and availability, and the method of near-patient glucose concentration measurement used. Separation of the effects of insulin infusion from those of euglycaemia could be achieved by comparing two groups receiving substantially different quantities of insulin, but achieving similar glycaemic control. However, mandatory continuous insulin infusion (to achieve higher insulin infusion rates than those required simply for glycaemic control) has not been undertaken in ICU patients for the prolonged periods utilized in studies of tight glycaemic control, although previous studies have shown much higher insulin rates to be safe in intensive care patients over short periods [7-9]. This trial aims primarily to determine if insulin infusions of 96 units/day can be achieved safely in a UK mixed general adult ICU, and what short-term biochemical effects occur with these infusions.

### Mechanisms by Which Insulin Might Alter Intensive Care Unit Mortality

Whilst it has long been known that chronic derangements of lipid profile are associated with long-term increases in mortality, more recent studies in the critical care environment suggest they also have an impact on acute survival [10]. The lipid profile of the critically ill patient is frequently abnormal, most commonly showing increased triglycerides combined with low levels of high-density lipoproteins (HDLs) and low-density lipoproteins (LDLs) cholesterol. This is partly due to the insulin resistance caused by acute illness or trauma. Observational studies suggest that the severity of these abnormalities correlate with an increased mortality [11].

By inhibiting hormone-sensitive lipase, insulin would be anticipated to decrease free-circulating triglycerides, which have been linearly correlated with mortality [12]. Excess triglycerides are thought to be toxic to the cell membrane and also increase oxygen consumption in an already ischemic environment.

Conversely, there is a body of animal work and some human studies that suggest that hypertriglyceridaemia is protective in gram negative sepsis because triglyceride-rich lipoproteins absorb endotoxin, thus preventing CD14 positive monocyte mediated cellular activation [13].

It may be that insulin, whilst reducing the amount of triglyceride available to absorb endotoxins, increases absorption by other mechanisms. It has been shown to ameliorate the decrease serum HDL levels in the critically ill. HDL has been shown to absorb the majority of endotoxins incubated with whole blood, and infusing reconstituted HDL has been shown to prevent endotoxin stimulated TNF-α production [14]. Moreover, HDL has been shown to provide endothelial protection by both antiapoptotic and antioxidative mechanisms [15].

Initially, this work seems contradictory to retrospective studies which showed a decreased mortality and incidence of sepsis in patients treated with long-term (lipid-lowering) 3-hydroxy-3-methyl-glutaryl-coenzyme A (HMG CoA) reductase inhibitors (statins) prior to their critical illness [16,17]. However, whilst reducing LDL cholesterol, statins, in common with insulin, cause moderate elevation of serum HDL [18]. The above mechanisms may therefore be similarly applicable, as statins are also known to have a range of other anti-inflammatory actions [19].

Critical illness is associated with increased protein catabolism that is relatively resistant to nutritional support [20]. The consequent loss of skeletal muscle leads to respiratory muscle weakness and a prolonged need for mechanical ventilation [21,22]. Small studies, mainly of burn patients, suggest insulin causes a decrease in protein catabolism, but have not shown morbidity or mortality benefits [23-25]. Work has suggested that the negative nitrogen balance that occurs is partly attributable to growth hormone resistance and decreased production and action of insulin-like growth factor 1 (IGF-1) [21]. Administration of high levels of growth hormone has been shown to improve nitrogen balance, but two large European randomized trials demonstrated increased mortality rates in the critically ill [26]. This was thought to be due to a combination of immunomodulatory and hyperglycaemic effects. Many of the effects of growth hormone are mediated via insulin-like growth factors (formerly somatomedins), modulation of which may avoid these unwanted effects.

Infusion of exogenous insulin into volunteers causes an increase in IGF-1 by inducing a reduction in the concentration of its binding protein insulin-like growth factor binding protein 1 (IGFBP-1) [27]. Serum IGF-1 levels have been consistently reported as low in acutely ill patients [28-31] and high IGFBP-1 levels have been associated with increased mortality in similar patients [32]. A longitudinal study of 18 heterogeneous critically ill patients showed initially low-circulating concentrations of IGF-1 and increasing IGF-1 values on recovery [33]. IGF-1 is both antiapoptotic and anabolic, and so might reduce the muscle wasting that leads to prolonged ventilator dependence. The vast majority of insulin-like growth factors in the circulation exist in a ternary complex with IGFBP-3 and an acid-labile subunit (ALS). This complex is too large to traverse the capillary endothelia and stimulate insulin or IGF receptors. ALS is also essential in sustaining plasma levels of IGF’s as it extends their half-life from 10 minutes to twelve hours. Levels of ALS are reduced in critical illness due to growth hormone and insulin resistance. The effect of mandatory insulin infusion on these aspects of the somatotrophic axis in the critically ill is unknown. Work from a subgroup of the original Van den Berghe trial has, somewhat surprisingly, shown a decrease in IGF-1 in the “tight glycaemic control” arm of the study [19].

The overall results of all these studies would seem to indicate that there may be a benefit from “tight glycaemic control”. A trial designed to clearly delineate insulin dose between two arms should provide more evidence for the science behind this new “therapy” if this feasibility trial can show that recruitment of ICU patients is possible and the therapy is shown to be able to be delivered safely.

### Objectives

The primary objective of the study is to determine the safety of mandatory insulin infusion in critically ill patients in a general ICU setting. This will be assessed by the number of episodes of hypoglycaemia and hypokalaemia per unit length of stay in intensive care.

The secondary objectives are to investigate four mechanisms by which exogenous insulin might alter outcome: (1) effects on lipid profiles (to include free fatty acids profiles, triglyceride, HDL, and LDL levels); (2) effects on nitrogen balance; (3) effects on the somatotrophic axis; and (4) effect on oxidative damage by measuring protein carbonyls.

## Methods

### Trial Design, Setting, and Patient Population

**Figure 1:**
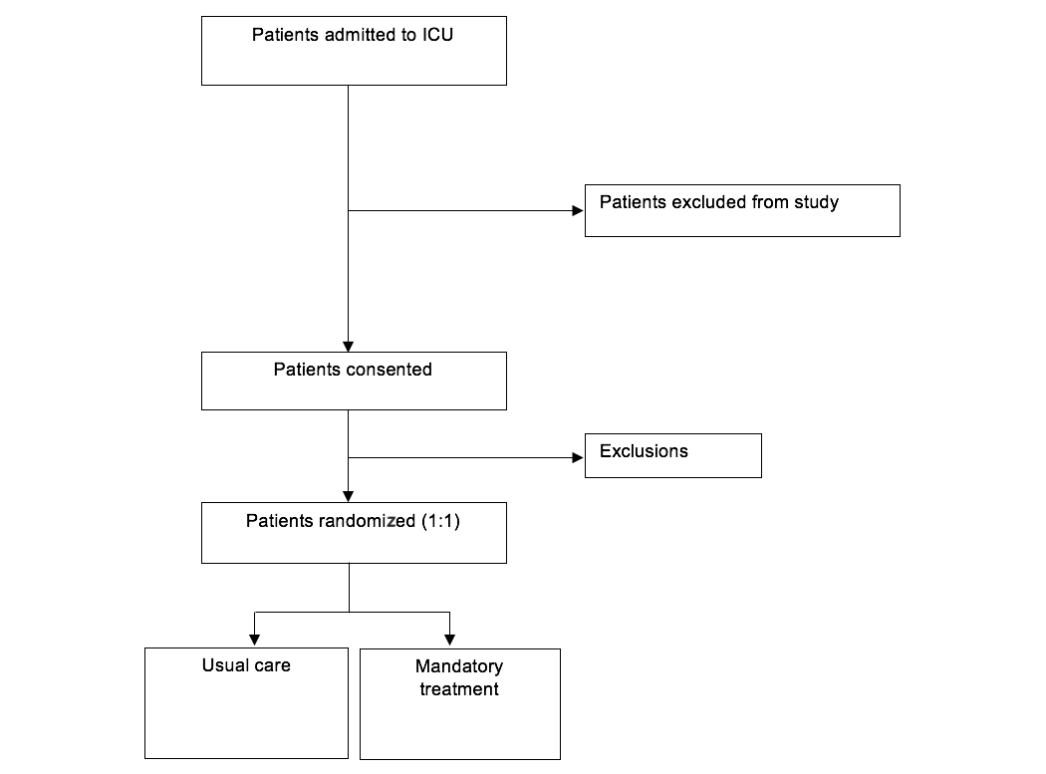
SPIRIT flowchart.

This is a randomized, controlled, open-label, single-centre trial comparing two strategies for the management of blood glucose in the ICU. The study will be conducted in one General (noncardiac) ICU at a UK university teaching hospital. All patients admitted to the ICU will be screened for eligibility. The study sponsor is the Oxford University Hospitals NHS Trust. A SPIRIT figure showing the schedule of enrolment, assessment of outcome measures, allocation, and interventions is shown in Figure 1. Those that were not diabetic, had not had hepato-biliary surgery or had a diagnosis other than pancreatitis, and who were expected to stay in the ICU for at least 48 hours were eligible for inclusion. The full inclusion and exclusion criteria is listed in Textbox 1.

**Inclusion and exclusion criteria.**

Inclusion criteria:

- 16 years or older
- Deemed by the attending physician to require 48 hours or more days of critical care treatment

Exclusion criteria:

- Known diabetes mellitus
- Admitted with diabetic ketoacidosis
- Current diagnosis of pancreatitis
- Undergone hepato-biliary surgery in the current admission
- Insulinoma or pituitary tumour
- Currently on, or likely to require, total parenteral nutrition
- Pregnant or breast-feeding
- Primary diagnosis of head injury
- Primary diagnosis of intracranial haemorrhage
- Primary diagnosis of stroke
- Inclusion in another study
- Currently placed under a section order
- Learning disability
- Unable to speak English and without a suitable translator
- Already on higher than 4 units of insulin per hour and have been so for at least 3 out of the last 24 hours
- No central line access
- Had cranial neurosurgery within the past 4 weeks
- Spent more than 24 hours on another ICU directly before this ICU admission
- Prisoners
- Undergone kidney-pancreas transplant in the current admission
- Failure to synthesise glucose

### Study Definitions

*Hypoglycaemia* is defined as a blood glucose measurement taken that is less than 2.2 mmol/L (this low level is set to be in agreement with other completed and in-progress trials [5,34-38]). *Hypokalaemia* is defined as a blood potassium measurement taken that is less than 2.6 mmol/L.

### Treatment

#### Line Management for Both Arms

A central line port should be dedicated to study use (as well as with Total Parenteral Nutrition). This port can be used for both insulin and glucose infusions. Both insulin and glucose infusions need priming (2 mls) for central infusion.

#### Potassium Measurement and Monitoring

Serum potassium should be monitored at least every 4 hours (by blood gas analysis or laboratory).

#### Mandatory Insulin Therapy (Intervention Arm)—4 Units of Actrapid Insulin Infusion per Hour

As soon as possible after randomization, an infusion of Actrapid (Novo Nordisk) should be prepared (50 units of Actrapid should be made up to 50 mls with 0.9% normal saline). This should be attached to an infusion pump to enable the patient to receive a mandatory insulin infusion of 4 units of Actrapid per hour. The syringe should be changed every 24 hours. A bag of 50% glucose should also be set up and the rate of this infusion is as per Table 1 to maintain normoglycaemia. Normoglycaemia will be maintained by sliding scale 50% glucose infusion with the rate of glucose infusion being dependent upon the blood glucose measurement as per Table 1.

If a patient has blood glucose measurements greater than 8 mmol/L after initial stabilisation (meaning the first 5 hours of treatment, or after initiation or change of feeding) on 3 successive occasions after stopping glucose infusion, they should be transferred to standard (insulin sliding scale) treatment. It should be checked that nutrition is not being given incorrectly prior to this decision being made.

**Table 1:** 50% glucose infusion rates for mandatory insulin therapy arm

Blood glucose measurement (mmol/L)	Glucose infusion rate
<2.6	Stop insulin infusion Bolus patient with 25ml of 50% glucose Seek medical advice Repeat blood glucose measurement in 30 minutes Once the blood glucose is back in the normal range the insulin infusion should be recommenced with the 50% glucose solution infusion rate increased by 8 mls per hour
<4.1	Increase 50% glucose infusion rate by 8 mls per hour Measure blood glucose again in 30 minutes
4.1 – 7	No change to infusion rate Measure blood glucose again in 60 minutes^a^
>7	Decrease 50% glucose infusion rate by 4 mls per hour Measure blood glucose again in 60 minutes^a^

^a^If blood glucose levels are changing rapidly, more frequent measurements may be necessary. If blood glucose levels remain in the desired range (and do not alter by more than 0.7 mmol/L each time) for 2 consecutive hours without alteration of glucose infusion, blood glucose measurement may be reduced to 2 hourly.

**Table 2:** Insulin infusion rates for usual care insulin therapy arm.

Blood glucose measurement (mmol/L)	Insulin infusion rate (units per hour)
0 – 4	0.5
4.1 – 7	1.0
7.1 - 11	2.0
11.1 – 17	4.0
17.1 – 27	7.0
>27	10.0

If changes to a patient's feed- or glucose-containing intravenous fluid regimes occur or if a feeding break occurs, hourly monitoring of blood glucose should be recommenced until the levels are again stable. Immediately after the stopping of feed, increase the glucose infusion rate by 8 mls per hour.

As soon as it is known a patient will be discharged the patient should have both glucose and insulin stopped. A blood gas (arterial or venous) should be taken just prior to departure to check that blood glucose and potassium levels are within the normal range.

#### Usual Care Insulin Therapy (Control Arm)

Only the frequency of blood glucose and potassium monitoring was changed for these patients (to be more frequent, every hour) and the standard unit guidelines for insulin administration should be followed. Actrapid should be made up as per the treatment arm. The unit guidelines are for the target range of 4-7 mmol/L blood glucose which should be achieved with insulin doses as per Table 2.

#### Therapy Administration

Once a patient is entered into the trial, either the patients’ attending nurse, the trial physician, or research nurse will set up the assigned therapy. The patients nurse will then follow either Table 1 or Table 2, depending upon allocation, to adjust the insulin and glucose infusions accordingly. As per the unit protocol and trial protocol, a physician should be called for any patients that has a blood glucose measurement of less than 2.6 mmol/L and the patient must be treated immediately for hypoglycaemia.

### Trial Protocol

#### Description of Trial Flow

Patients will be identified in the ICU through twice daily surveillance by the research coordinator or treating ICU physicians. Each patient’s eligibility will be verified by the use of a screening form that summarizes the inclusion and exclusion criteria. The patient needs to have been randomized within 24 hours of their admission to the ICU.

#### Outcomes

The primary outcome is to determine the number of episodes of hypoglycaemia and hypokalaemia, and compare this with the usual treatment (control) group.

Additional outcome measures used to determine safety will be duration of mechanical ventilation, duration of ICU and hospital stay, hospital mortality, and measures of renal, hepatic and haematological dysfunction. Antibiotic use will be used as a surrogate marker for nosocomial infections. Biochemical markers as specified in the protocol will also be measured to compare between groups.

#### Sample Size Justification

The study duration will be limited by funding, so a formal power calculation has not been performed. Instead, a recruitment target of 120 patients is set. It was felt that as the recruiting ICU had admitted 912 patients in the 12 months prior to the start of the trial, of which 270 stayed for 5 or more days this would be a realistic recruitment target.

#### Informed Consent

Wherever possible, informed consent will be obtained from patients prior to randomization. It is recognized that in the majority of cases the patients will be unable to give informed consent due to alterations in level of consciousness caused by illness and therapeutic sedation. Although a relative cannot provide consent on behalf of a patient whose consent cannot be obtained, a written agreement stating no objection will be obtained from the patient’s relative. The relative will be given the information sheet making them aware that they can only offer an opinion as to whether they know if the patient would not have objected to taking part in medical research.

In the event that the relative cannot be spoken to in person, a verbal “no objection” will be obtained by telephone and randomization will be undertaken. Verbal assent will be obtained by the recruiting consultant or an individual with appropriate experience he/she nominates. The quality of consent will be ascertained from the responses given by the relative. Questions will be encouraged and the relative will have the opportunity to clarify any of the information given. Signed confirmation of the verbal assent will be sought, retrospectively, if practical.

Once the patient regains the capacity to comprehend the details of the study, they will be asked for permission to include their data in the study. If a patient or relative refuses consent/assent then the patient will receive the usual treatment as defined by the clinician responsible for the patient's care.

If a patient dies before regaining consciousness and the relative has given assent, the patient's data will be included in the study. The person taking consent will ensure that the patient (or the relative) receives a copy of the Consent/No Objection Form, and that a copy is placed in the patient’s notes. The original will be filed in the Trial Office. This process is in line with the Mental Capacity Act 2005 and has been approved by a UK Research Ethics Committee.

#### Randomization

Allocation to a treatment arm will be made randomly. A Web-based specialist randomization service will be used (www.thesealedenvelope.com). Randomization will occur once a patient meets the inclusion criteria and has either consented to the trial or his/her relative (Personal Legal Representative) has provided verbal or written assent. When a member of the trial team logs onto the randomization service, basic descriptive information will be requested. The allocation will be minimized according to the patient’s gender. The requirement to titrate an infusion of insulin in one group and an infusion of glucose in the other group, using two different schedules, precludes blinding.

#### Data Collection

Clinical data will be collected in a standardized way on a trial specific data form. Data will be transcribed from the patient’s notes or the clinical information system (Philips CareVue) by members of the PERMIT research team. Baseline measures of severity of illness (Acute Physiology and Chronic Health Evaluation II score, Sequential Organ Failure Assessment score) will be collected on admission, along with the demographics required to demonstrate equivalent groups.

All estimations of blood glucose and serum potassium, whether performed in the central laboratory or using near patient testing, will be collected from the clinical information system (CareVue). The doses of insulin, potassium, glucose, and antibiotics will also be collected. Nitrogen input will be estimated from the documented feed rates. Blood glucose levels will be monitored hourly, and 3-hydroxybutrate levels daily, using the Abbott system.

Blood and urine samples will also be taken from all randomized patients whilst they remain in the ICU. Specifically, blood samples will be taken at baseline (Day 1), Day 3, Day 5, Day 7 and Day 14. Urine collections will be taken over the preceeding 24-hour period on Days 3, 5, and 7.

Data are collected for the entire period of the patient’s stay in ICU. The hospital discharge date will be obtained from the local patient administration system (PAS) by the Trial Office. When a patient is transferred to another acute hospital, the discharge date will be requested from the receiving hospital. To enable collection of patient status 30 days after randomization, the PAS will be interrogated to determine the patient’s location at 30 days. For those patients that have been discharged, the patient’s General Practitioner will be contact by telephone to establish their location. There is no planned long-term follow up.

#### Statistical Analysis

The principal comparisons will be between those allocated to usual care and those allocated to mandatory insulin (the “intention to treat principle”). The primary outcome variable will be the differences in the number of hypoglycaemic and hypokalaemia events between groups which will be compared by chi-squared tests. Differences between multiply measured continuous variables will be compared using analysis of variance. To allow for differences in length of stay between patients, wherever possible, variables will be converted to time-weighted averages if they are used as a single, between-group measure.

#### Data Safety and Monitoring

Data relating to the safety of patients will be reviewed by an independent statistician (Dr Tony Brady) once 40 patients have been randomized to the trial. The data reviewed will specifically relate to: (a) serious unanticipated events (SAEs) and (b) deaths at 30 days (any cause)

#### Ethical Considerations

This trial protocol was approved by a research ethics committee (Oxfordshire Local Research Ethics Committee C) (REC REF: 05/Q1606/103) and by the UK Medicines Healthcare Regulatory Agency as competent authority (CTA No. 21439/0207/ 001-0001). The protocol adheres to principles of the Declaration of Helsinki and Good Clinical Practice.

## Results

The project was funded in 2005 and enrolment was completed 2007. The trial was suspended after recruiting 109 patients and all patients were followed up as per this protocol. Data analysis is currently underway and the first results are expected to be submitted for publication in 2018. 

## Discussion

This protocol for a randomized controlled trial will allow further investigation of the impact of mandatory insulin infusions and determine if a powered trial would be feasible. The trial aims to determine if the insulin is the biological effector that alters mortality in these patients. This requires similar blood glucose control in two groups of patients, but a different insulin dose. This can be achieved with a mandatory insulin infusion in one group and an “as needed” insulin infusion in the other, with a variable glucose infusion in the mandatory group to maintain euglycaemia. This should allow us to determine the safety of mandatory insulin infusions of this dose in this patient group. Secondly, we aim to determine whether insulin or euglycaemia is the biological effector that alters outcome to inform and plan future research. Euglycaemia in the absence of additional insulin may be ineffective in reducing mortality and so the routine adoption of “tight” glycaemic control, as is happening at present, will be largely ineffective.

This study should allow us to further interpret the Van den Berghe study as their study design necessarily resulted in two differences between the arms, the blood glucose level and the insulin dose. The authors linked the reduction in mortality to changes in the blood glucose level; in essence proposing that hyperglycaemia per se has some ill effect that increases the chance of death, and so elevated blood glucose carries an attributable mortality. The biological pathway for this was thought to be related to impaired leukocyte function in the presence of high glucose concentrations. However, the proposal that maintenance of normoglycaemia led to the benefits seen by improving immune function is belied by the relatively small difference in median glucose values between the two groups compared with the levels of plasma glucose required in previous studies to cause immune dysfunction (39, 40).

There are potential risks from this study as a mandatory insulin infusion will have two main effects that might lead to patient harm: an excessive reduction in blood glucose level and an excessive reduction in serum potassium concentration. In a modern ICU with an appropriate staffing ratio and close monitoring, the risk of both of these events would be expected to be low. In the Van den Berghe study 5.1% of the patients in the “tight” glucose control group had one or more blood glucose determination of less than 2.2 mmol/l. The number of episodes of hypokalaemia were not reported.

## Abbreviations

ALS: acid-labile subunit
HDL: high-density lipoprotein
ICU: intensive care unit
IGF: insulin-like growth factor
IGFBP: insulin-like growth factor binding protein
LDL: low-density lipoprotein
PAS: Patient Administration System

## Appendix

Multimedia Appendix 1 SPIRIT checklist.
