# Supplementary material for: Outcome of Critically ill Patients Undergoing Mandatory Insulin Therapy Compared to Usual Care Insulin Therapy: Protocol for a Pilot Randomized Controlled Trial
Source: JMIR Res Protoc. 2018 Mar 8;7(3):e44. doi: 10.2196/resprot.5912 (PMC5865000; doi:10.2196/resprot.5912)
Supplement: Multimedia Appendix 1 [file resprot_v7i3e44_app1.pdf]

Figure 1. SPIRIT 2013 figure showing schedule of enrolment, assessments, allocation and intervention for each participant. (* only taken if still an inpatient in ICU)

	Enrolment	Allocation	Study period		
**TIMEPOINT**	**$-t_1$**	**0**	***Time in ICU***	***Hospital discharge***	***30 days***
**ENROLMENT:**					
**Eligibility screen**	X				
**Consent**	X				
**Intervention Allocation**		X			
**INTERVENTIONS:**					
Mandatory Insulin Therapy or Usual Care Insulin Therapy			X		
**ASSESSMENTS:**					
Blood glucose monitoring			X		
Potassium monitoring			X		
APACHE II score			X		
Nitrogen input			X		
Antibiotics administered			X		
Insulin administered			X		
Glucose administered			X		
Potassium administered			X		
3-hydroxybutrate levels			X		
Blood samples for ELISA analyses (Day 1, 3, 5, 7 and 14)*			X		
Urine samples for ELISA analyses (Day 3, 5 and 7)*			X		
Discharge date			X	X	
Mortality status			X	X	X

Location				X	X
